# Persistent memory as an effective alternative to random access memory in metagenome assembly

**DOI:** 10.1186/s12859-022-05052-8

**Published:** 2022-11-30

**Authors:** Jingchao Sun, Zhining Qiu, Rob Egan, Harrison Ho, Yue Li, Zhong Wang

**Affiliations:** 1 MemVerge Inc, Milpitas, CA 95035 USA; 2 Department of Energy Joint Genome Institute, Berkeley, CA 94720 USA; 3 School of Natural Sciences, University of California at Merced, Merced, CA 95343 USA; 4 Environmental Genomics and Systems Biology Division, Lawrence Berkeley National Laboratory, Berkeley, CA 94720 USA

**Keywords:** Metagenome assembly, Persistent memory, Out-of-memory

## Abstract

**Background:**

The assembly of metagenomes decomposes members of complex microbe communities and allows the characterization of these genomes without laborious cultivation or single-cell metagenomics. Metagenome assembly is a process that is memory intensive and time consuming. Multi-terabyte sequences can become too large to be assembled on a single computer node, and there is no reliable method to predict the memory requirement due to data-specific memory consumption pattern. Currently, out-of-memory (OOM) is one of the most prevalent factors that causes metagenome assembly failures.

**Results:**

In this study, we explored the possibility of using Persistent Memory (PMem) as a less expensive substitute for dynamic random access memory (DRAM) to reduce OOM and increase the scalability of metagenome assemblers. We evaluated the execution time and memory usage of three popular metagenome assemblers (MetaSPAdes, MEGAHIT, and MetaHipMer2) in datasets up to one terabase. We found that PMem can enable metagenome assemblers on terabyte-sized datasets by partially or fully substituting DRAM. Depending on the configured DRAM/PMEM ratio, running metagenome assemblies with PMem can achieve a similar speed as DRAM, while in the worst case it showed a roughly two-fold slowdown. In addition, different assemblers displayed distinct memory/speed trade-offs in the same hardware/software environment.

**Conclusions:**

We demonstrated that PMem is capable of expanding the capacity of DRAM to allow larger metagenome assembly with a potential tradeoff in speed. Because PMem can be used directly without any application-specific code modification, these findings are likely to be generalized to other memory-intensive bioinformatics applications.

## Background

Ever since the first 454/Solexa sequencers broke the dawn of the next-generation sequencing revolution, the rate of increase in sequencing data has been growing exponentially at a pace exceeding Moore’s law. The number of nucleotide base pairs (bp) in public repositories is estimated to reach the exabase scale ($10^{18}$ bp) before 2025 [20]. Metagenomics is one of the main contributors to this rapid growth of data. Metagenomics, the study of microbial genomes isolated directly from their natural habitat, frees researchers from the need for laborious and time-consuming isolating and culture of microbes [21, 22]. Powered by next-generation sequencing, metagenomics offers an unprecedented opportunity to gain a deep understanding of the microbial communities around us or within us and to harness their genetic and metabolic potential for our health and environmental safety.

However, the construction of individual microbial genomes from a complex microbial community with thousands of species from billions of short reads faces both data and algorithmic challenges (reviewed in [2]). It was initially thought impossible until pioneering work demonstrated its feasibility [5, 8]. At that time, assemblers developed for single genome assembly were used for metagenome assembly because there were no metagenome-specific assemblers available. Since then, metagenome assemblers have been developed that consider the specific characteristics of metagenomic datasets, such as uneven sequencing depth for different member species. These assemblers include meta-IDBA [15], metaSPAdes [13], MEGAHIT [9], and many others. Several recent studies have provided a comprehensive comparison of the computational performance and accuracy of these assemblers [12, 18, 23]. While most of these assemblers can efficiently take advantage of the modern CPU’s multiple processing capabilities, they are limited on a single computer node and, therefore, are not able to assemble very large datasets due to the limited memory capacity. For terabase-scale metagenome datasets, researchers have very few options. Swapping memory using fast disks or even from multiple machines over a fast network running JumboMem [14] can help if the extra memory required is minimal, but this significantly extends runtime. meta-RAY [3] uses MPI to distribute a large metagenome assembly to multiple computer nodes. To overcome its limitation that it only assembles very abundant species, hybrid strategies have been developed to first use meta-RAY in a computer cluster to assemble abundant species (which often comprise most of the sequencing data), followed by MEGAHIT or metaSPAdes in a single node to assemble unassembled reads [24]. Recently, MetaHipMer used UPC++ to assemble very large metagenome datasets with high accuracy and efficiency [6], but it runs best on a supercomputer that is not readily available to most researchers.

New algorithms have the potential to dramatically reduce the memory requirement for metagenome assembly. For example, MEGAHIT uses a data structure called the succinct de Bruijn graph that significantly reduces memory consumption [9]. Since new algorithms take a long time to develop, a more straightforward strategy is to expand the memory capacity of a single system, which does not involve application-specific software development and can be generically applied to other memory-intensive applications. The XSEDE large shared memory system, Blacklight, contains 16 TB of shared memory that allows extremely large-scale genome assemblies [4]. A drawback of this system is that it costs tens of millions of dollars to build. As the price of PC DRAM (DDR4) becomes more affordable, many computer systems are built with several terabytes of DRAM. Intel® Optane™ Persistent Memory (PMem) is a new type of RAM that is packaged in DDR4-compatible modules of 128 GB, 256 GB, and 512 GB capacities, much larger than typical DDR4 modules currently available (16 GB, 32 GB, and 64 GB) [7]. Intel’s Persistent Memory is twice the capacity of the current largest available DDR4 module (256 GB). And at each DIMM capacity point, PMEM is half to one-third the cost of DRAM [11] , which is promising for memory-intensive applications such as metagenome assembly.

PMem can be configured in one of two modes. “Memory Mode” is volatile and uses the DRAM in the system as a cache to improve the performance of the PMem, which has a random access latency approximately four times higher than that of DRAM. This effectively decreases the total RAM available. For example, if a machine is equipped with 192 GB DRAM and 1 TB PMem, software tools can only address 1 TB available memory in the “Memory Mode”. The 192 GB DRAM will be ‘hidden’ and will be used exclusively as a cache for the PMem. Another limitation of “Memory Mode” is that it provides a fixed DRAM/PMem ratio for all applications without flexibility. An application can use PMem in Memory Mode without code modification. In “AppDirect Mode”, the PMem is nonvolatile, and both DRAM and PMem are addressable as system memory. In the above example, “AppDirect Mode” enables users to use the 192 GB DRAM plus the 1 TB PMem. It also enables resuming from software crashes as data on PMEM is persistent. However, applications must be refactored to be able to use this mode, which is time-consuming and expensive. MemVerge’s Memory Machine™ is a software tool that runs in the user space on Linux systems to virtualize system memory. It is based on the “AppDirect Mode” but has two main advantages over the “AppDirect Mode”. First, it allows users to configure specific DRAM/PMem ratios for each individual application. For example, applications accessing a larger amount of “hot data”, or frequently accessed data, can benefit from a larger DRAM/PMem ratio so that hot data are cached in DRAM. Second, it enables any application to access PMEM without code modifications by automatically remaps memory pages so that “hot” data is moved to DRAM and “cold” data are moved to PMem, which may also result in overall performance.

PMem has been successfully applied to many memory-intensive applications, such as databases [1, 10, 16, 17]. This work reports its first application on metagenome assemblies on a server configured with DRAM and PMem. We evaluated the feasibility and performance of the running time and memory consumption of several common metagenome assemblers.

## Results and discussion

### Detailed memory usage and CPU profiling of metagenome assembly with metaSPAdes

**Fig. 1:**
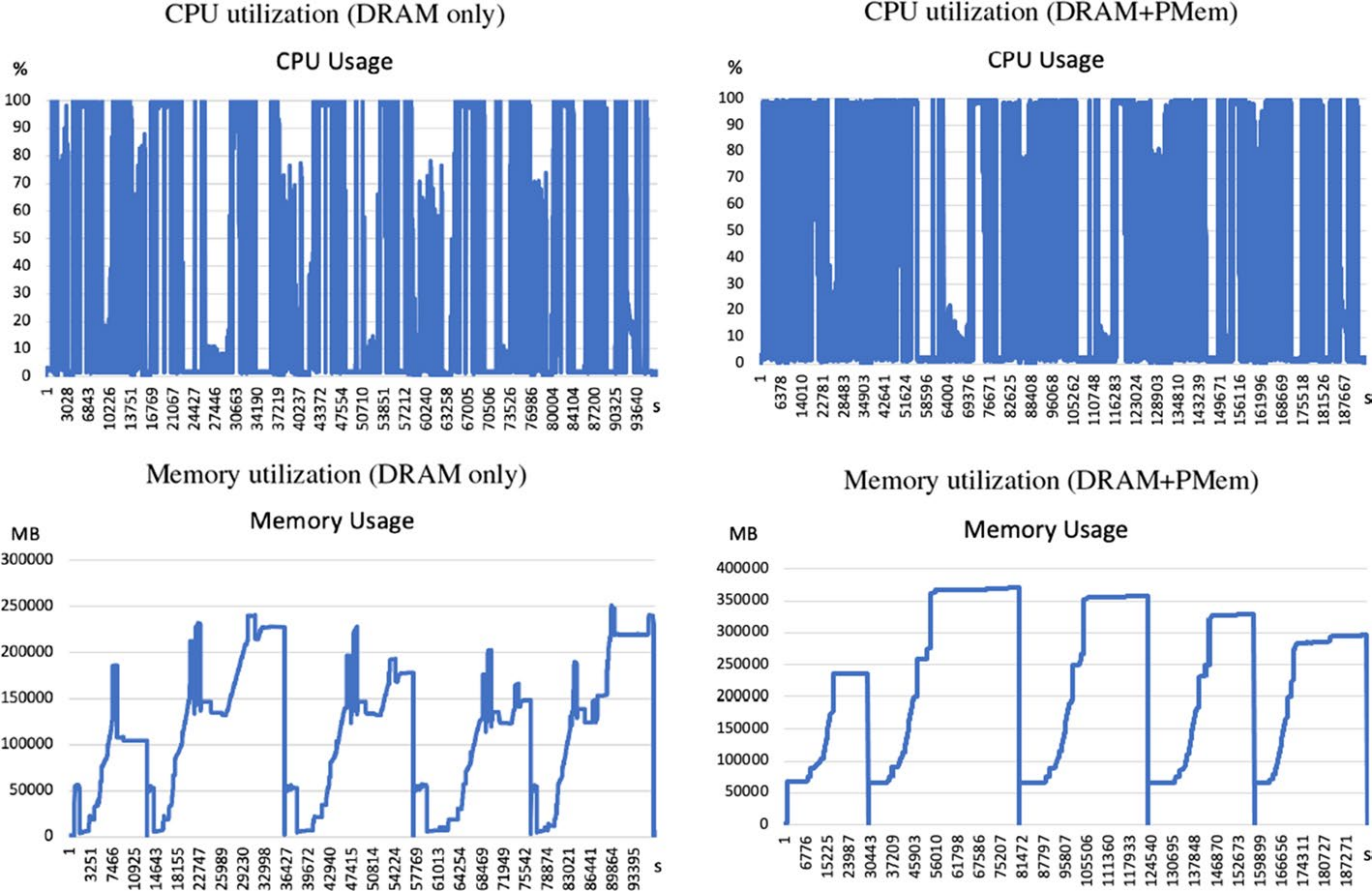
System metrics for metaSPAdes execution on the Wastewater Metagenome dataset using DRAM-only (left) or using PMem with 32 GB DRAM tiering (right). System metrics were recorded for the period that metaSPAdes was running. The CPU utilization timeline (in percentage), memory usage (in MB) are shown. The horizontal axis shows the wall clock time in seconds. The running time of DRAM + PMem was 2.04 times that of DRAM-only

Modern metagenome assemblers take a “multi-k” approach to assemble species of various abundances in the microbial community. For example, the metaSPAdes pipeline first constructs an initial de Bruijn graph, then iteratively simplifies it one k-mer size at a time. Then it transforms the graph into the assembly graph, followed by graph simplification and graph traversal to obtain contigs [13]. To profile its use of computational resources, we ran metaSPAdes on the 233 GB Wastewater metagenome dataset and record its CPU, memory utilization. As expected, each phase of de Bruijn graph construction is computationally and memory intensive, as well as the final assembly graph phase (Fig. 1). In any phase where the memory consumption of metaSPAdes is greater than the available DRAM, an Out-of-Memory (OOM) failure occurs. To ensure the success of the pipeline, a workstation must have sufficient memory that is larger than the peak (maximum) memory.

To test whether or not enabling PMem leads to changes in CPU or memory utilization, we used the Memory Machine to configure 32 GB of DRAM while supplying the rest with PMem. We saw an increase in peak memory consumption from (250 GB to 370 GB), which is likely due to the overhead incurred by the Memory Machine software, as the page size was increased from 4 KB to 2 MB. The general pattern of memory utilization was the same. Furthermore, CPU utilization increases due to additional hot-swapping options (Fig. 1).

### PMem can effectively satisfy the requirement of metaSPAdes for large amount of DRAM at a small cost of running speed

**Fig. 2:**
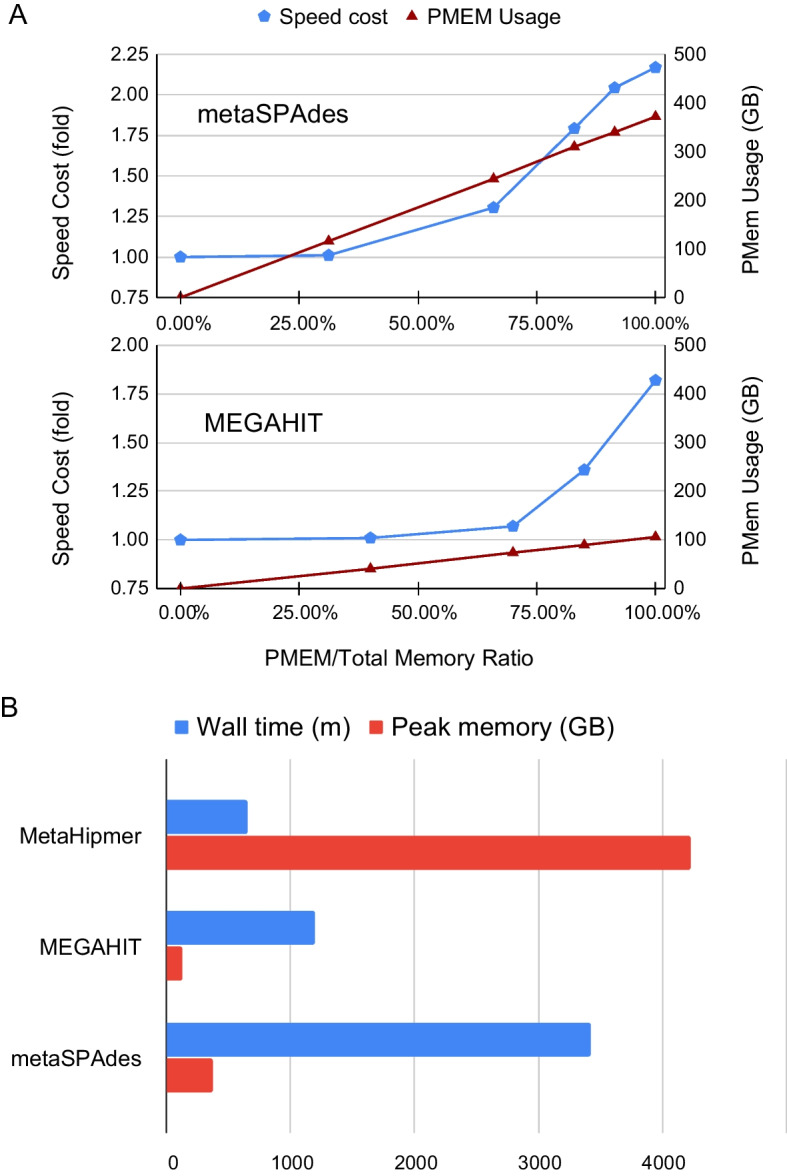
**A** Memory (red) and speed (blue) metrics of running metaSPAdes (top) and MEGAHIT (bottom) on the Wastewater metagenome dataset. The percentage of PMem of the total memory (DRAM+PMem) is shown on the horizontal axis, while the speed cost in folds is shown on the left vertical axis, whereas the amount of PMem used in GB is shown on the right vertical axis. For each memory configuration, we ran the pipeline twice and showed the average results (speed and PMem amounts). **B** Comparison of MetaHipMer2, MEGAHIT, and metaSPAdes running on the Wastewater metagenomics dataset. All three assemblers were run using 100% PMem. The total time of the wall clock (in minutes) is shown in blue, and the maximum memory consumption (in GB) is shown in red

The maximum memory consumption to run metaSPAdes on the Wastewater Metagenome dataset with only DRAM was approximately 250 GB, and the run was finished in 26.3 hours. To test whether or not PMem can be used to substitute DRAM for metaSPAdes, we used the Memory Machine to configure decreasing amounts of DRAM and measured PMem usage and running time. We found that PMem can substitute DRAM in all tested memory configurations, resulting in savings in DRAM of up to 100% (Fig. 2A, top). Meanwhile, increasing DRAM savings also led to a longer execution time of metaSPAdes due to the slower performance of PMem compared to DRAM. As shown in Fig. 2A, top, substituting up to 30% of the total memory with PMem led to no appreciable slowdown, but after that the slowdown became apparent. When 100% PMem is used to replace DRAM, the run time of the metaSPAdes on the Wastewater Metagenome dataset was approximately twice as long ($2.17\times$). In all configurations, the total amount of memory consumption (DRAM + PMEM) is largely the same at 372 GB, except in the DRAM-only case (250 GB).

The longer running time in PMem is probably caused by a combination of slower PMem writing performance and the cost of swapping data between DRAM and PMem. Memory Machine does provide a software development kit (SDK) to precisely allocate memory on DRAM or PMem based on each application to reduce the data migration cost, but doing so would require additional software engineering effort.

Running metaSPAdes on the larger 1.2 TB Antarctic Lake Metagenome Dataset with only DRAM led to an OOM error, as the required memory exceeds the total amount of DRAM (768 GB). When running with Memory Machine with 550 GB DRAM tiering, the peak memory reached 1.8 TB, and the run was completed successfully in 11.15 days.

### PMem supports other metagenome assemblers including MetaHipMer2 and MEGAHIT

In addition to metaSPAdes, we also tested MetaHipMer2 [6] and MEGAHIT [9] on the Wastewater Metagenome dataset. As shown in (Fig. 2A, bottom), with 100% PMem, the run time of MEGAHIT on the Wastewater Metagenome dataset was $1.75\times$ that on DRAM, and the run time increases as PMem to total memory ratio increases. In contrast, the run time of MetaHipMer2 on the Wastewater Metagenome dataset for different PMem ratios were roughly the same: 807.6 (min), 792.97 (min), 788.45 (min), and 675.85 (min) for 97%, 98%, 99%, and 100% PMem to total memory ratio, respectively. The slightly better performance with more Pmem is probably due to the slightly decreased data migration cost. When the amount of DRAM is a small fraction of total memory (MetaHipMer2 used 4.16 TB of memory), more DRAM leads to more data migration.

MetaHipMer2 consumed the most memory (peaked at 4.2 TB), while it took the least time (647 min). MEGAHIT used the least memory (peaked at 124 GB), but took almost twice as long and finished the assembly in 1191 min. metaSPAdes used a peak memory of 372 GB and took the longest time (3423 min). The comparison of the three assemblers is shown in Fig. 2B.

We did not attempt to compare the three assemblers using the Antarctic Lake Metagenome Dataset because MetaHipMer2 would likely run into OOM. It required 13 TB of RAM distributed across multiple nodes on a supercomputer to complete this dataset in a previous experiment.

## Conclusions

For terabyte-scale metagenome assembly projects, existing solutions are either expensive (a fat shared-memory machine) or have limited hardware availability (supercomputers). We demonstrated the feasibility of running a large-scale metagenome assembly on commodity hardware by substituting DRAM with persistent memory (PMem). If a running time is not a critical factor, we showed that PMem is a cost-effective option to extend the scalability of metagenome assemblers without requiring software refactoring, and this likely applies to similar memory-intensive bioinformatics solutions.

## Methods

### Hardware environment

The MEGAHIT and MetaHipMer2 experiments in the first paragraph in Section 2.3 were carried out on a single server with 2 Intel(R) Xeon(R) Platinum 8260L 2.40 GHz, each with 24 cores. Its memory includes a total of 192 GB DDR4 DRAM and 12 × 512 GB PMem 100 series (6 TB total). One 2.5 TB SSD were used. The server was running CentOS 8 Linux with a 4.18.0-193.19.1.el8_2.x86_64 kernel. The rest of the experiment were carried out on a single server with 2 Intel(R) Xeon Gold 6248 3.0 GHz (Turbo 3.9 GHz), each with 24 cores (48 threads). Its memory includes a total of 768 GB DDR4 DRAM and 12 × 512 GB PMem 100 series (6 TB total). Six 2 TB SSDs were configured as a single 12 TB volume. The server was running CentOS 8 Linux with a 4.18.0-305.7.1.el8_4.x86_64 kernel. It is worth noting that PMem requires newer Intel CPUs, and a full list of supporting CPUs can be found at https://www.intel.com/content/www/us/en/support/articles/000058038/memory-and-storage/intel-optane-persistent-memory.html.

### Software environment

Memory Machine Release 2.1 by MemVerge Inc. was used to provide memory virtualization. The normal memory page size in the Linux kernel is 4KB. Transparent HugePages (THP) is a memory management system in the Linux kernel that tries to use HugePages (2MB) greater than the default. Using large page sizes can improve system performance by reducing the amount of system resources required to access page table entries. Memory Machine implements its own management of HugePages, and thus the default THP was disabled. Memory Machine can be launched by running the command mm before other commands.

The DRAM, and CPU usage was monitored by the unix commands “free”, and “ps”, respectively. The DRAM tiering and PMEM usage was obtained by “mvmcli show-usage” (a tool provided by MemVerge Memory Machine Release 2.1).

### Metagenome assemblers

SPAdes (Saint Petersburg Genome Assembler) version 3.15.3 was used. For using with Memory Machine, the source code was compiled using dynamic linking by turning off the options SPADES_STATIC_BUILD and SPADES_USE_MIMALLOC in the make file. Furthermore, the OpenMP scheduling was changed from dynamic to static (Line 255 in /src/common/utils/kmer mph/kmer index builder.hpp). The following metaSPAdes command line options were used “-only-assembler”, “-k 33,55,77,99,127”, “-meta”, “-t 96”.

MetaHipMer2 (MHM2) version 2.1.0.37-g01c2b65 was used. The source code for MHM2 was compiled with UPC++[upcxx-ipdps19] version 2021.3.0, which in turn was built using the included ‘install_upcxx.sh’ script that builds UPC++ using the default ‘smp’ conduit for Symmetric Multi-Processor and shared-memory for communication between processes. MHM2 was executed using the default options on the dataset: ‘mhm2.py -r filtered_wgs_fastq.fastq’

MEGAHIT version v1.2.9 was used. The source code was downloaded and compiled following the instructions on https://github.com/voutcn/megahit.

## Data Availability

(1) The Wastewater Metagenome Dataset was downloaded from NCBI (BioProject: PRJNA506462, Accession: SRR8239393). It was derived from samples taken at a waste water treatment plant in Idaho [19]. This dataset has a total uncompressed size of 164.8 GB. (2) The Antarctic Lake Metagenome Dataset was downloaded from the JGI GOLD database with the accession no Gs0118069 (https://gold.jgi.doe.gov/study?id=Gs0118069). It was derived from samples taken from two meromictic lakes in Antartica [24]. This dataset has twelve files totalling 1.37 TB.

## Abbreviations

OOM: Out-of-memory
Pmem: Persistent Memory
DRAM: Dynamic random access memory
THP: Transparent HugePages
SSD: Solid state disk
MHM2: MetaHipMer v2

## References

[1] Aerospike. Building real-time database at petabyte scale. 2019. https://www.intel.com/content/www/us/en/architecture-and-technology/optane-persistent-memory-database-restart-demo.html.

[2] Ayling M, Clark MD, Leggett RM (2020). New approaches for metagenome assembly with short reads. Brief Bioinform.

[3] Boisvert S, Raymond F, Godzaridis É, Laviolette F, Corbeil J (2012). Ray meta: scalable de novo metagenome assembly and profiling. Genome Biol.

[4] Brian Couger M, Pipes L, Squina F, Prade R, Siepel A, Palermo R, Katze MG, Mason CE, Blood PD (2014). Enabling large-scale next-generation sequence assembly with blacklight. Concurr Comput: Pract Exp.

[5] Hess M, Sczyrba A, Egan R, Kim T-W, Chokhawala H, Schroth G, Luo S, Clark DS, Chen F, Zhang T (2011). Metagenomic discovery of biomass-degrading genes and genomes from cow rumen. Science.

[6] Hofmeyr S, Egan R, Georganas E, Copeland AC, Riley R, Clum A, Eloe-Fadrosh E, Roux S, Goltsman E, Buluç A (2020). Terabase-scale metagenome coassembly with MetaHipMer. Sci Rep.

[7] Intel. Product brief. 2020. https://www.intel.com/content/dam/www/public/us/en/documents/product-briefs/optane-persistent-memory-200-series-brief.pdf.

[8] Iverson V, Morris RM, Frazar CD, Berthiaume CT, Morales RL, Armbrust EV (2012). Untangling genomes from metagenomes: revealing an uncultured class of marine Euryarchaeota. Science.

[9] Li D, Luo R, Liu C-M, Leung C-M, Ting H-F, Sadakane K, Yamashita H, Lam T-W (2016). MEGAHIT v1. 0: a fast and scalable metagenome assembler driven by advanced methodologies and community practices. Methods.

[10] MemVerge. SQL databases and memory management. 2021. https://memverge.com/wp-content/uploads/2021/03/White-Paper_SQL-Databases-and-Memory-Management%E2%80%8B.pdf. Accessed March 2021.

[11] MemVerge. More memory less cost. 2022. https://memverge.com/more-memory-less-cost/.

[12] Meyer F, Fritz A, Deng Z-L, Koslicki D, Gurevich A, Robertson G, Alser M, Antipov D, Beghini F, Bertrand D, et al. Critical assessment of metagenome interpretation-the second round of challenges. bioRxiv. 2021. 10.1038/s41592-022-01431-4 PMC9007738 35396482

[13] Nurk S, Meleshko D, Korobeynikov A, Pevzner PA (2017). metaSPAdes: a new versatile metagenomic assembler. Genome Res.

[14] Pakin S, Johnson G. Performance analysis of a user-level memory server. In: Proceedings of the 2007 IEEE international conference on cluster computing (cluster 2007), Austin, Texas; 2007. p. 249–58.

[15] Peng Y, Leung HC, Yiu S-M, Chin FY (2011). Meta-IDBA: a de novo assembler for metagenomic data. Bioinformatics.

[16] Redis. Intel Optane persistent memory and Redis. 2019. https://www.intel.com/content/www/us/en/architecture-and-technology/optane-pmem-redis-enterprise-ra.html.

[17] SAPHANA. Persistent memory improves SAP HANA. 2019. https://aerospike.com/partners/intel-optane/.

[18] Sczyrba A, Hofmann P, Belmann P, Koslicki D, Janssen S, Dröge J, Gregor I, Majda S, Fiedler J, Dahms E (2017). Critical assessment of metagenome interpretation—a benchmark of metagenomics software. Nat Methods.

[19] Stalder T, Press MO, Sullivan S, Liachko I, Top EM (2019). Linking the resistome and plasmidome to the microbiome. ISME J.

[20] Stephens ZD, Lee SY, Faghri F, Campbell RH, Zhai C, Efron MJ, Iyer R, Schatz MC, Sinha S, Robinson GE (2015). Big data: astronomical or genomical?. PLoS Biol.

[21] Tyson GW, Chapman J, Hugenholtz P, Allen EE, Ram RJ, Richardson PM, Solovyev VV, Rubin EM, Rokhsar DS, Banfield JF (2004). Community structure and metabolism through reconstruction of microbial genomes from the environment. Nature.

[22] Venter JC, Remington K, Heidelberg JF, Halpern AL, Rusch D, Eisen JA, Wu D, Paulsen I, Nelson KE, Nelson W (2004). Environmental genome shotgun sequencing of the Sargasso Sea. Science.

[23] Vollmers J, Wiegand S, Kaster A-K (2017). Comparing and evaluating metagenome assembly tools from a microbiologist’s perspective-not only size matters!. PLoS ONE.

[24] Wang Z, Ho H, Egan R, Yao S, Kang D, Froula J, Sevim V, Schulz F, Shay JE, Macklin D, et al. A new method for rapid genome classification, clustering, visualization, and novel taxa discovery from metagenome. bioRxiv; 2019. p. 812917.

